# Editorial: Digital biomarkers in movement disorders

**DOI:** 10.3389/fneur.2025.1600018

**Published:** 2025-05-08

**Authors:** Christopher D. Stephen, Federico Parisi, Martina Mancini, Carlo Alberto Artusi

**Affiliations:** ^1^Department of Neurology, Massachusetts General Hospital and Harvard Medical School, Boston, MA, United States; ^2^Department of Physical Medicine and Rehabilitation, Motion Analysis Laboratory, Spaulding Rehabilitation Hospital and Harvard Medical School, Charlestown, MA, United States; ^3^Department of Neurology, Oregon Health & Science University, Portland, OR, United States; ^4^Department of Neuroscience ‘Rita Levi Montalcino', University of Torino, Torino, Italy

**Keywords:** movement disorders, digital biomarkers, motion analysis, digital health, patient perspective

## Introduction

There is a substantial gap in the assessment of movement disorders in the clinic, and clinical trials, with the current gold standard involving clinical rating scales performed by expert clinicians. This not only limits access but these rater-dependent measures are time-consuming, lack sensitivity to disease progression, have ceiling effects in advanced disease, and floor effects in the early stages (1). In addition, for therapeutic and disease-modifying clinical trial readiness in movement disorders, there are increasing calls for sensitive and rater-independent, multi-modal biomarkers, including quantitative digital motor biomarkers to quantify the motor examination, identify the earliest signs of disease manifestation, and obtain a fine-grained monitoring of disease progression (2–5). Such measures could be deployed remotely (6, 7), increasing access, particularly in underserved regions, and reducing the sample size, with consequent reduction of time and costs. Such measures are particularly important in rare or combined movement disorders, where sample sizes are small, and the presence of overlapping features and phenomenology make clinical assessment especially challenging. To overcome such obstacles, objective measures of motor performance using digital technology are currently being studied, and such measures are now being included in early-adopting clinical trials (8).

## Patients' perspectives and use feasibility

Firstly, patient perspectives on the use of digital technology are of vital importance to ensure data clinically relevant and important to patients is collected, and as buy-in from patients is essential for effective deployment (9, 10). To this end, Paccoud et al. performed a large-scale patient survey regarding the willingness of people with Parkinson's disease (PD) to adopt and engage with digital devices. They found a high level of willingness to use digital technology and acceptance of data sharing. This study further emphasizes the importance of having a patient-centered focus for deploying digital technology and highlighting differences in preferences across the age range. Further, Evers et al. sought to identify the perspectives of patients and healthcare providers (physiotherapists, nurses, and neurologists) regarding personalized monitoring of PD symptoms. They also conducted focus groups of these groups, and interviews with neurologists, comparing currently used monitoring tools to wearable sensors. Barriers included wanting to avoid focusing on symptoms of PD, and lack of an easy-to-use tool. Importantly, they identified a mismatch between priorities in patients and those of providers (which varied considerably by specialty), highlighting that personalized, patient-centered strategies will be important in the future.

In tandem with digital assessment of motor symptoms and movement analysis, digital patient-reported outcome measures, including digital diaries to assess motor fluctuations and disease progression in people with movement disorders such as PD, are important as clinical and research tools (11). Asai et al. compared an electronic diary to a standard paper diary assessing motor fluctuations. Electronic diaries were faster and showed a greater degree of correlation with patient-reported measures of disease severity, suggesting that electronic diaries may be more accurate than paper diaries in reflecting motor fluctuations in PD.

Wearable sensors, including those for continuous monitoring of mobility in daily life, are a burgeoning field in the assessment of movement disorders (12–14), with considerable interest in PD (15). Antonini et al. describe the results of two multi-site clinical studies assessing the performance and wearability of a system called PDmonitor. The system includes five inertial measurement unit (IMU) sensors to attach to both wrists and ankles and across the waist. They assessed meaningful aspects of wearable sensor use, including acceptable wearability of the device. Measurements assessing bradykinesia, gait, tremor, freezing of gait, dyskinesias, and on/off states correlated with clinical evaluations, suggesting the feasibility of assessing PD motor symptoms. Acceptability of the technology was good, as well as compliance. Interestingly, the study indicated that the monitoring device worn on the waist seemed to be more inconvenient compared to devices worn on other body parts.

Telemonitoring systems can be used to continuously monitor patients with movement disorders (16) over long periods, and for potential eligibility assessment of therapies (17). Konitsiotis et al. performed a telemonitoring study in 17 people with PD using a mobile app and five wearable sensors to measure everyday activities and digital reported outcomes over a 2-year time period. Telemonitoring positively impacted motor symptom control and enhanced patient satisfaction, which could improve adherence to treatment plans.

## Gait assessment in the laboratory and in daily life

Gait analysis is a common research tool for the assessment of gait disorders, including PD (18). A marker-based infrared camera setup represents the gold standard for gait analysis. However, this approach can only be performed in a specialized gait laboratory, and hence, video-based assessment has evolved over time (19). Yin et al. used a markerless integrated camera system, including an RGB and depth camera, to perform 3D gait analysis. They compared early-stage PD patients to controls and used machine-learning approaches. Several typical features distinguished early-stage PD from controls, an integrated analysis accurately identified PD, and machine-learning algorithms predicted clinical scores.

Shah et al. compared people with PD with falls and those without falls, using three inertial sensors. They created models to predict future fall risk, with the most consistent predictive features being gait variability, particularly variability of the toe-out angle of the foot, as well as turning domains, including pitch angle during mid-swing and peak turn velocity.

## Combination of multiple digital technology systems

Furthermore, digital technology systems (3, 20) can also be used in combination. Debelle et al. used multi-component digital technologies to collect mobility and medication data and to assess feasibility. They assessed people with PD over 7 days with a single IMU applied to the lower back to assess digital mobility outcomes, a smartphone to contextualize data, a smartwatch to assess self-reported medication adherence, and a diary to track motor complications, as well as a usability questionnaire. They suggested the feasibility of their approach, with the IMU and smartphone being usable, although there were issues with the smartwatch, both technical and related to tremor, or not feeling reminder vibrations, as well as a lack of familiarity with the system, indicating potential limitations.

As an attempt to operationalize digital health approaches (21), Alberts et al. sought to apply digital technologies together as the Waiting Room of the Future for PD, which could be deployed into the clinic and integrated into the electronic health record. Their PD-Optimize paradigm involves digital assessments completed on an iPad of motor function (manual dexterity and walking speed, a digital adaptation of the 10 m walking test) and cognitive aspects (visual memory and processing speed), combined with patient-reported outcomes. They describe the development and integration of their platform into clinical practice. Insights from the clinical use of PD-Optimize led to the development of a virtual reality platform to evaluate instrumental activities of daily living in PD patients.

## Atypical Parkinsonism and other movement disorders

Digital technology has also been applied to atypical Parkinsonism, with comparison to PD, and as potential markers of disease progression (22, 23). Dale et al.reviewed the use of multiple modalities assessing gait and balance (force plates, 3D motion capture, and inertial sensors) and exercise interventions in progressive supranuclear palsy (PSP). They describe cross-sectional studies using wearable sensors comparing PSP to PD and longitudinal studies assessing PSP, and their limitations. They suggest potential practical applications, including abnormal anticipatory posture and the use of wearable sensors for longitudinal assessment, which may be useful for clinical trials.

Robertson-Dick et al. performed a first study of gait analysis in fragile X-associated tremor/ataxia syndrome (FXTAS). FXTAS has a wide clinical spectrum including tremor, ataxia and Parkinsonism. Digital measures have sought to identify features of prodromal disease in FXTAS (24). The authors used digital gait markers to compare patients with FXTAS, PD, and essential tremor (ET) using six IMUs under various gait conditions, and an instrument Timed Up and Go, in addition to cognitive assessments. Metrics differentiated PD from FXTAS and ET but none distinguished FXTAS from ET, and suggested that future study may aid in accurate and timely diagnoses.

Posturography using force plates is a long-established method to assess static and dynamic balance in vestibular disorders (25) and movement disorders (26–30). Bao et al. used static and dynamic posturography and compared PD and multiple system atrophy (MSA) of the Parkinsonism (MSA-P) and cerebellar (MSA-C) types. While static posture was similar between groups, all dynamic posturography parameters differentiated MSA from PD, with worse postural control in the medial-lateral direction. MSA patients had a greater degree of worsening with the eyes closed condition.

The simple use of spiral drawing is a useful assessment for clinically distinguishing different movement disorders (31), with increasing research interest in digital automated analysis (32) and particularly for the severity assessment of tremor disorders (33), such as ET (34). Toffoli et al. compared patients with PD to controls using a smart ink pen and utilizing machine learning for classification. PD patients had reduced fluency, with smoothness, correlating with clinical scores, and lower, more variable applied force, with accurate classification of PD compared to controls.

Musician's dystonia is a debilitating occupational dystonia, which has received little research assessing motor physiology (35, 36). Sata et al. take an uncommon case study of musician's dystonia involving the lower extremities of a drummer, and used electromyography of lower extremity muscles to assess bass drum pedaling and performed muscle synergy analysis using non-negative matrix factorization. This revealed shared muscle synergies in data with and without dystonic movement. Spatially, there was dystonia-specific muscle synergy, hypothesized to be related to compensatory movement, while temporally there was earlier over activation in timing, considered related to the dystonic movements.

## Conclusion

We are at the threshold of the accepted use of digital biomarkers to assess movement and motor disorders in isolation or as a combined platform and their integration into clinical practice (37). In addition to a growing literature on sensor-based assessment, there is also the potential for automated video analysis using computer vision (38, 39). Such approaches could aid in early diagnosis (including in prodromal stages), promote more accurate and earlier differential diagnosis, and track patient symptoms over time. These advantages hold the potential for more accurate clinical assessment, which benefits clinical care and research, and may lower sample sizes, time, and eventually costs of clinical trials.
